# Middle School Students Effectively Improve Stroke Knowledge and Pass Them to Family Members in China Using Stroke 1-2-0

**DOI:** 10.3389/fneur.2020.00203

**Published:** 2020-04-08

**Authors:** Xutong Li, Yang Liu, Amey Vrudhula, Renyu Liu, Jing Zhao

**Affiliations:** ^1^Department of Neurology, Minhang Hospital, Fudan University, Shanghai, China; ^2^Department of Anesthesiology and Critical Care, Perelman School of Medicine at the University of Pennsylvania, Philadelphia, PA, United States

**Keywords:** school-based intervention, pre-hospital delay, stroke, emergency medical service, stroke educational tool

## Abstract

**Background and Purpose:** This study tests the hypothesis that middle school and high school students can improve their stroke knowledge using Stroke 1-2-0, a stroke educational tool, and pass this knowledge on to their family members.

**Methods:** A total of 625 students and 198 parents/grandparents participated in learning about stroke using Stroke 1-2-0. After a group training session for the students by a neurologist at school, the students took educational material to home and educated their parents/grandparents. A questionnaire was given to students, parents/grandparents before, immediately after, and 1 year after the educational event.

**Results:** All participants agreed that Stroke 1-2-0 was a much easier tool to remember than FAST. Almost all the students (96.4%) remembered the meaning of Stroke 1-2-0 as compared to 7.3% from the base line (p < 0.001). The rate of complete Stroke 1-2-0 mastery from 96.3% fell to 84.4% at 3 months and 63.8% at 1 year after training (p < 0.001). Following education from children, the proportion of parents/grandparents who mastered Stroke 1-2-0 was significantly higher than baseline (79.9 vs. 24.8%).

**Conclusion:** Middle school and high school students can effectively use Stroke 1-2-0 to improve their stroke knowledge and pass this knowledge to their family members. Sustained educational efforts and repeated educational events are needed though.

## Introduction

Stroke is one of the leading causes of death and disability worldwide, especially in developing countries. Current guidelines recommend that recombinant tissue plasminogen activator be administered within 4.5 h of stroke onset in the treatment of acute ischemic stroke (1, 2). However, only 1.6% of patients with ischemic stroke in China received thrombolytic therapy, and the median prehospital delay time for stroke patients was 15 h in urban areas (3). Thus, “FAST,” the stroke awareness program used in English-speaking countries, has had limited effectiveness since it has been used to improve stroke awareness in China over a decade period. Existing data show a weak correlation between the knowledge of FAST and the utilization of the emergency medical system (EMS) (4, 5). Poor EMS utilization, 18.9%, among stroke patients is observed in China (6). To overcome existing language barriers and improve EMS utilization, we created the Stroke 1-2-0 educational program by linking the stroke symptoms defined in FAST to 1-2-0, China's medical emergency phone number. This work was recently presented in *Lancet Neurology* (7). Since this strategy uses only numbers, it should be easily conveyed regardless of significant language barriers. We have demonstrated that Stroke 1-2-0 successfully improved the stroke knowledge deficiency in community physicians (8). The effectiveness of improving stroke awareness in junior high school students and the successful passing of this knowledge on to their family members have been demonstrated using FAST (4). This study tests the hypothesis that middle and school students can effectively and easily improve their stroke knowledge using Stroke 1-2-0 and pass this knowledge on to their parents and grandparents in China.

## Methods

The Ethics Committee of Minhang Hospital, affiliated with Fudan University, approved this investigation. Since no patient information was involved in this investigation, no informed consent was needed. Students in one middle school and one high school in Shanghai were initially surveyed in 2016 before and after the stroke awareness education and re-surveyed at 3 months and 1 year later. A total of 613 valid questionnaires were completed before training, 566 questionnaires were completed immediately after the training, 539 were completed 3 months later, and 478 valid questionnaires were completed a year later. The questions contained in the questionnaire are listed in Tables 1, 2. The educational material distributed to students included a cartoon describing our Stroke 1-2-0 strategy and a 1-min educational video that we published recently on how to recognize stroke and act immediately to dial 1-2-0 (7). Each student received 20 min of on-site PowerPoint-based stroke educational training provided by a neurologist in a class (a group setting, not an individual setting). The educational training included stroke identification, first aid, the specific content of Stroke 1-2-0, the content of FAST, and a cartoon along with a 1-min educational video.

**Table 1:** The students completing questionnaire.

	**Baseline**		**Post-education**		***P***	**After 3 months**		***P***	**After 1 year**		***P***
	***n***	**%**	***n***	**%**	**value**	***n***	**%**	**value**	***n***	**%**	**value**
(1) Do you know how to quickly identify a stroke? (Only one answer can be chosen)											
1. Yes	103	16.8	546	96.5	0.000^*^	474	87.9	0.000^*^	365	76.4	0.000^*^
2. I don't know, but I want to know	452	73.7	14	2.5	0.000^*^	39	7.2	0.000^*^	80	16.7	0.000^*^
3. Don't know, don't want to know	58	9.5	6	1.1	0.000^*^	26	4.8	0.003^*^	33	6.9	0.151
(2) What do you do if you see someone who might have an acute stroke? (More than one answer can be chosen)											
1. Call family and friends	138	22.5	93	16.4	0.010^*^	135	25.1	0.331	169	35.4	0.000^*^
2. Tell him to take a break.	60	9.8	14	2.5	0.000^*^	20	3.7	0.000^*^	44	9.2	0.757
3. Call 120 ambulance	540	88.1	544	96.1	0.000^*^	486	90.2	0.298	438	91.6	0.058
4. Don't know	30	4.9	5	0.9	0.000^*^	22	4.1	0.57	6	1.3	0.001^*^
(3) Do you remember the specific content of Stroke 1-2-0? (Only one answer can be chosen)											
1. Completely correct	45	7.3	545	96.3	0.000^*^	455	84.4	0.000^*^	305	63.8	0.000^*^
2. Partially correct	109	17.8	8	1.4	0.000^*^	49	9.1	0.000^*^	124	25.9	0.001^*^
3. Complete error	459	74.9	13	2.3	0.000^*^	35	6.5	0.000^*^	49	10.3	0.000^*^
(4) FAST and stroke 1-2-0, which one do you think is easier to remember by Chinese? (Only one answer can be chosen)											
1. FAST	102	16.6	53	9.4							
2. Stroke 1-2-0	397	64.8	487	86							
3. Don't know or other	114	18.6	26	4.6							

*P-values are obtained from Fisher exact test compared with baseline*.

* *indicates statistically significant*.

**Table 2:** The parents and grandparents completing questionnaire.

	**Baseline**		**Post-education**		***P***	**After 1 year**		***P***
	***n***	**%**	***n***	**%**	**value**	***n***	**%**	**value**
(1) Do you know how to quickly identify a stroke? (Only one answer can be chosen)								
1. yes	88	44.4	158	90.8	0.000^*^	44	62.0	0.013^*^
2. I don't know, but I want to know	104	52.5	12	6.9	0.000^*^	23	32.4	0.004^*^
3. Don't know, don't want to know	6	3.0	4	2.3	0.756	4	5.6	0.298
(2) What do you do if you see someone who might have an acute stroke? (More than one answer can be chosen)								
1. Call family and friends	14	7.1	5	2.9	0.097	18	25.4	0.000^*^
2. Tell him to take a break.	9	4.6	3	1.7	0.15	6	8.5	0.234
3. Call 120 ambulance	175	88.4	162	93.1	0.154	63	88.7	0.937
4. Don't know	7	3.5	7	4	1	3	4.2	0.727
(3) Do you remember the specific content of Stroke 1-2-0? (Only one answer can be chosen)								
1. Completely correct	49	24.7	139	79.9	0.000^*^	31	43.7	0.004^*^
2. Partially correct	26	13.1	7	4	0.003^*^	22	31.0	0.002^*^
3. Complete error	123	62.1	28	16.1	0.000^*^	18	25.4	0.000^*^
(4) FAST and stroke 1-2-0, which one do you think is easier to remember by Chinese? (Only one answer can be chosen)								
1. FAST	18	9.1	13	7.5				
2. stroke 1-2-0	165	83.3	155	89.1				
3. Don't know or other	15	7.6	6	3.4				

*P-values are obtained from Fisher exact test compared with baseline*.

* *indicates statistically significant*.

**Figure 1:**
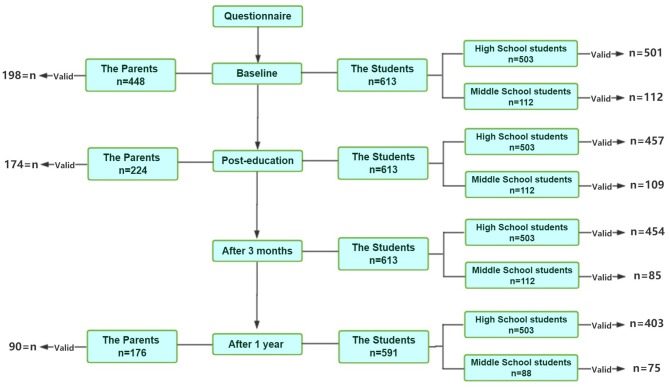
Flowchart of the study. Valid in the charts represents the valid questionnaires collected.

In order to demonstrate that this information could be easily transmitted by even middle school students, the middle school students who participated in the education session were asked to educate their parents and grandparents about what they learned in the training session and asked to hand over the same questionnaire to their parents and grandparents and collect the questionnaire after completion. Each student was given two questionnaires to take home. The parents completed a questionnaire before receiving education from the children and then completed the same questionnaire after the education. The pre-training and post-training questionnaires were completed on site. At 1 year later, the students took home the same questionnaire, and the parents did it again without any additional education effort. A total of 198 valid questionnaires were completed before training, 174 questionnaires were completed immediately after the training, and 90 valid questionnaires were completed a year later (Figure 1).

## Statistical Analysis

The IBM SPSS Statistics software was used for statistical analysis (Version 22.0, IBM Corporation, Armonk, New York, USA). The data are presented as either an actual number or a percentage. The comparison between the groups was based on Fisher exact test. A *P* < 0.05 was considered to indicate a significant difference.

## Results

### Assessment of Students

We presented a flowchart for the participants and the valid questionnaires collected in Figure 1. A total of 625 students (112 middle school and 513 high school students) participated in this investigation. There were 613 (98%) valid questionnaires collected before the training, 566 (91%) immediately after the training, 539 (86%) at 3 months after the training, and 478 (76%) at 1 year after the training (Table 1). Before the training, 73.74% of the students did not know how to quickly identify stroke but expressed their desire to learn. Following the training, a substantially higher percentage of students thought that they knew how to recognize stroke compared to baseline (96.5 vs. 16.8%). The percentage of students who knew how to recognize stroke remained high compared to baseline but dropped to 87.9% at 3 months and to 76.4% at 1 year after the training, respectively. Complete mastery of Stroke 1-2-0 content also significantly improved after the training (96.3 vs. 7.3%). It dropped to 84.4% at 3 months and 63.8% at 1 year later after the training.

### Assessment of Students' Parents and Grandparents

The 112 middle school students who participated in the Stroke 1-2-0 educational lesson were asked to give a stroke education lesson to their parents/grandparents and subsequently collect the questionnaires from them. A total of 224 parents/grandparents participated in the study, and 198 and 174 valid questionnaires were collected before and after the children explained the Stroke 1-2-0 to their parents/grandparents. As indicated in Table 2, More than 80% of the participants thought Stroke 1-2-0 was more suitable than FAST for members of the Chinese population. Before training, 52.5% of participants did not know how to quickly identify stroke. After the explanation, 90.8% of the participants thought that they now knew how to quickly identify stroke. After 1 year, this proportion dropped to 62.0%. Complete mastery of Stroke 1-2-0 also significantly improved after the training, from 24.5 to 79.9%. However, it dropped to 44% at 1 year later.

## Discussion

As predicted, Stroke 1-2-0 is more acceptable than FAST as an educational tool in China. Most of the students and their parents effectively mastered the educational tool and remembered the signs and symptoms of stroke through the short 20-min education activity. Although the rate of correct responses to questions decreased over time, it was still significantly above baseline (prior to training) at both 3 months and 1 year following the training.

While stroke mainly occurs in the elderly, younger family members, especially young school-going students, are often the first witnesses. Therefore, rapid recognition of stroke symptoms by younger family members is critical. This study demonstrated not only that students could be educated to master a stroke recognition tool but also that they could be an alternative task force to educate their parents/grandparents about identifying stroke using Stroke 1-2-0.

Students have multiple advantages as an alternative task force for stroke education. They have similar educational environments and strong learning abilities. Through group training at school, students can significantly improve their stroke awareness and pass this knowledge on to their parents and grandparents using FAST as a tool (9). This is the first educational study using Stroke 1-2-0 with a 1-year follow up for both the students, their parents, and their grandparents in China. Over time, some loss on the awareness of stroke is completely normal. Interestingly, a sharper decline in Stroke 1-2-0 mastery was observed in parents and grandparents. People's memory gradually declines with time. This study found that the knowledge mastery dropped significantly after 1 year. Based on this, it is suggested that sustained educational efforts are needed for this portion of the population. Biannual educational sessions may obtain the optimal educational outcome. Stroke can occur at any age, and stroke in the young poses social and economic consequences to society (10). Educational activities at school could potentially improve the students' awareness in improving lifestyle, reducing stroke risk factors and identifying stroke quickly.

The major limitation of this study is that this is a relatively small-scale study in a highly selective school district in Shanghai where the program was vigorously promoted; thus, bias could exist to affect the results and their interpretations. Only the parents of middle school (but not high school) children were included in the study. The parents' educational levels were not assessed. However, these results remain encouraging. Transmitting stroke awareness to older family members by relating knowledge through the schoolchildren could serve as a model for similar, larger-scale studies across China. This school-based educational strategy was proposed in a recent summary statement on potential strategies in reducing stroke prehospital delay (11).

In conclusion, this study successfully shows that middle and high school students can use Stroke 1-2-0 to effectively and easily improve their stroke knowledge and pass such knowledge on to their family members in China. However, sustained educational efforts and repeated educational events are needed especially in the older people due to significant fading of knowledge over time.

## Data Availability Statement

The datasets generated for this study are available on request to the corresponding author.

## Ethics Statement

The studies involving human participants were reviewed and approved by the Ethics Committee of Minhang Hospital, affiliated with Fudan University, Shanghai, China. Written informed consent to participate in this study was provided by the participants' legal guardian/next of kin.

## Author Contributions

XL performed data collation and statistics, article editing, and proofreading. YL helped data collation and article proofreading. AV helped data analysis, paper writing, and editing. RL proposed the study, assisted the study design, performed data analysis, paper writing, and editing. JZ designed and supervised the study, performed data analysis, and paper writing.

### Conflict of Interest

The authors declare that the research was conducted in the absence of any commercial or financial relationships that could be construed as a potential conflict of interest.
